# Optimized Open-Source Setting for Subjecting Rodents to Chronic Normobaric Hypoxia in Facilities with Minimal Nitrogen Supply

**DOI:** 10.3390/life16071140

**Published:** 2026-07-09

**Authors:** Jorge Otero, Miguel A. Rodríguez-Lázaro, Raffaella Salama, Daniel Mbanze, Gorka Solana, Vicent Muñoz-Vaño, Yolanda Cámara, Isaac Almendros, Ramon Farré

**Affiliations:** 1 Unit of Biophysics and Bioengineering, School of Medicine and Health Sciences, University of Barcelona, Casanova 143, 08036 Barcelona, Spain; m.rodriguez@ub.edu (M.A.R.-L.); raffaellasalama@gmail.com (R.S.); isaac.almendros@ub.edu (I.A.); 2 CIBER of Respiratory Diseases (CIBERES), Monforte de Lemos 3–5, 28029 Madrid, Spain; 3 Faculdade de Engenharias e Tecnologias, Universidade Save, Estrada Nacional N/C. Agostinho Neto Nhaguiviga, Maxixe, Inhambane, Mozambique; garteche@unisave.ac.mz; 4 Research Group on Neuromuscular and Mitochondrial Diseases, Vall d’Hebron Research Institute, Universitat Autònoma de Barcelona, Passeig Vall d’Hebron 119–129, 08035 Barcelona, Spain; vicent.munoz@vhir.org (V.M.-V.); yolanda.camara@vhir.org (Y.C.); 5 CIBER of Rare Diseases (CIBERER), Monforte de Lemos 3–5, 28029 Madrid, Spain

**Keywords:** oxygen sensor, animal research, gas concentration control, CO_2_ monitoring, hypoxia model

## Abstract

Very prevalent respiratory and cardiovascular diseases result in chronic hypoxia, promoting metabolic, kidney, heart, and other malignant diseases. Hypoxia research employs animal models based on chronically breathing hypoxic air (O_2_ < 21%), usually by injecting N_2_ into the animal’s chamber. However, continuous high-flow N_2_ supply is available only in limited facilities, reducing the capability for hypoxia research to be widely conducted. Here, we describe an optimized setting for subjecting rodents to chronic normobaric hypoxia by requiring minimal N_2_ supply. The primary aim of this study was the technical development and optimization of a system for chronic normobaric hypoxia exposure rather than testing a specific biological hypothesis The setting is based on providing the O_2_ consumed by the animals and eliminating the exhaled CO_2_ and water vapor. O_2_, CO_2_, temperature, and humidity in the hypoxic chamber are controlled by an Arduino-based unit activating a pump that introduces room air to restore the metabolized O_2_. Another pump continuously recirculates the chamber air through a Peltier-based dryer and CO_2_-absorbing soda lime. To correct any deviation in the actual value of hypoxia within the chamber, the control unit allows the injection of N_2_ into the chamber from a gas source. The setting performance was successfully tested in vivo when subjecting mice to 11%–O_2_ chronic hypoxia. This device, requiring a low N_2_ supply, may facilitate in vivo experimental research on hypoxia-related diseases.

## 1. Introduction

Respiratory and cardiovascular diseases are nowadays among the most prevalent conditions [1,2], and their high morbidity and mortality are expected to increase further because of the current worldwide epidemic of obesity [3,4] and the rise in life expectancy [5,6]. Owing to either poor air oxygenation in the lungs or inadequate blood circulation and distribution, these diseases usually result in chronic hypoxia, a state of poor oxygenation of the cells in tissues and organs. Hypoxia severely affects normal cell function, resulting in negative consequences in multiple organs, such as myocardial ischemia, metabolic diseases, chronic heart and kidney diseases, reproductive diseases, and cancer [7]. Hence, hypoxia plays a relevant pathophysiological role in human health and is a subject of intense investigation [8].

Research on the mechanisms involved in the multiorgan consequences of normobaric hypoxia requires animal models based on chronically breathing air with O_2_ concentration below the usual 21% of atmospheric air. The most common experimental setting for achieving hypoxia in animal models is placing them in a chamber where the O_2_ concentration in the ambient air is reduced by injecting a flow of N_2_ [9,10]. Indeed, regulating the flow of room air and of N_2_ entering the chamber is the simplest way to control the concentration of O_2_ that the animals breathe. However, a relatively high flow of N_2_ is required, since to prevent hypercapnia, the injected gas must sufficiently wash out the CO_2_ exhaled by the animals.

In most cases, the source of N_2_ required for the most conventional setting to continuously subject animals to hypoxia cannot be based on conventional bottles of compressed gas because of the high consumption required. A possible option would be a N_2_ generator (based on N_2_ extraction from room air by a pressure swing adsorption concentrator). However, these devices are expensive, limiting their use for this application. The most common alternative is a centralized N_2_ gas pipeline system installed in the building to provide gas to different points of use. However, this infrastructure, which is commonly available in hospitals and cell biology laboratories, is usually unavailable in most animal laboratory facilities. Such requirements for a continuous N_2_ source limit the widespread extension of hypoxia-related in vivo research.

To facilitate research employing hypoxic animal models in facilities not having access to a continuous high-flow N_2_ source, we aimed to design, build, and test an open-source, low-cost device requiring minimal N_2_ provision. The aim of the present study was the technical development of the device and was not animal research to test a biological hypothesis.

## 2. Materials and Methods

**Figure 1:**
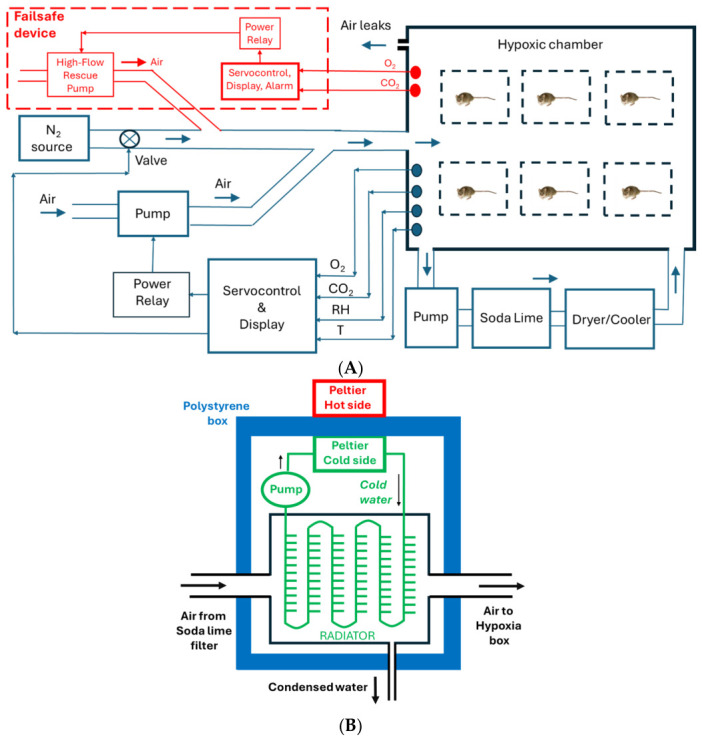
(**A**) Diagram of the hypoxia setting for mice. (**B**) Dryer/cooler detail. The hypoxia chamber accommodates conventional mouse cages. O_2_ and CO_2_ concentration, relative humidity (RH) and temperature (T) in the air chamber are continuously measured by sensors placed inside the chamber. A servocontrol circuit uses the sensor signals to automatically regulate the target gas concentrations by activating the injection of N_2_ and room air as required. A closed circuit of air circulation within the chamber, consisting of an air pump, a soda lime reservoir and an air dryer/cooler, allows absorption of the CO_2_, water vapor and heat produced by mouse metabolism. A completely independent failsafe device, with an independent power relay, sensors, air pump and control unit, is indicated in red. A diagram of the dryer/cooler is presented at the bottom. The air from the hypoxia chamber is dried and refrigerated by circulating it through a water radiator cooled with a Peltier unit, and then injected into the hypoxia chamber. See text for explanation.

Contrary to most conventional settings for producing hypoxic air by continuous N_2_ injection, the device described here aims to minimize the supply of N_2_ when subjecting mice to controlled chronic hypoxia. The setting is based on ensuring the balance of the gases involved in mice metabolism, thus providing the O_2_ consumed and eliminating the exhaled CO_2_ and water vapor. A schematic description of the setting is presented in Figure 1: The O_2_ and CO_2_ concentrations, relative humidity (RH), and temperature (T) inside the hypoxic chamber are continuously measured by sensors, and processed by the control and display unit, based on an Arduino microcontroller (Arduino, Ivrea, Italy).

The O_2_ concentration was measured using an electrochemical oxygen sensor, SEN0322 (DFRobot, Shanghai, China), while CO_2_ concentration, temperature (T), and relative humidity (RH) were measured using a Grove—CO_2_, Temperature and Humidity Sensor, 101020952 (Seeed Studio, Shenzhen, China).

A high-resistance air leak connects the hypoxic chamber with the atmospheric air. The air is continuously recirculated within the chamber by the use of a domestic aquarium membrane-based pump (60 L/min) (Hailea, Chaozhou, China). A soda lime filter is used to absorb the generated CO_2_. Medical-grade soda lime should be used since it contains ethyl violet, a pH-sensitive dye which changes from colorless to violet as the soda lime absorbs CO_2_. A dryer/cooler (based on an AC 12 V 120 W liquid-cooling thermoelectric Peltier-based unit providing cool water that circulates through an air radiator) is used to regulate the temperature and to condense water vapor (Figure 1, bottom). For this purpose, the dryer/cooler section is enclosed into a thermally isolating box made with polystyrene walls including an outlet to extract the condensed water (Figure 1, bottom).

A second domestic aquarium pump (30 L/min) (Hailea, Chaozhou, China) is used to introduce room air into the hypoxic chamber to supply the O_2_ consumed by the mice. Ideally, the setting would not require any additional N_2_ injection to keep a target level of hypoxia since the consumption of O_2_ and the production of CO_2_ and water vapor are balanced by the components of the setting (Figure 1). However, the control unit allows the injection of N_2_ into the chamber from a gas bottle through an electrically controlled valve (DFRobot, Shanghai, China).

Figure 1 includes (in red) a completely independent (and optional) failsafe device to protect the animals against accidental life-threatening hypoxia or hypercapnia occurring in case any component (e.g., power, pumps, sensors, microprocessors) of the hypoxia device fails. The failsafe device has its own power source, O_2_ and CO_2_ sensors and Arduino control loop. The gas concentrations are continuously sensed and displayed, and in case concentration of O_2_ is below 8% or CO_2_ is above 2000 ppm, both light and sound alarms are activated, and a high-flow (60 L/min) air pump (similar to the ones described for the hypoxic device) is activated to flush room air into the chamber. Using the failsafe device is not strictly required for the normal function of the hypoxic setting, but it is strongly recommended. To avoid confusion, the technical description of the independent failsafe device is provided in a specific folder of the Supplementary Materials.

**Figure 2:**
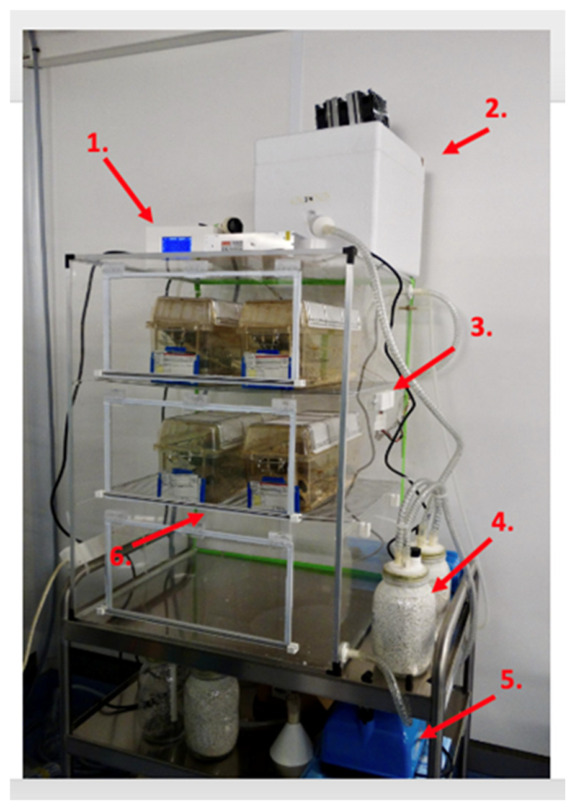
Picture of the implemented hypoxic setting with capacity for 30 mice (only 4 conventional mouse cages shown). The main components are indicated by numbers: (1) control and display unit based on an Arduino microcontroller; (2) dryer–cooler unit; (3) CO_2_, O_2_, temperature (T), and relative humidity (RH) sensors; (4) soda lime canister; (5) air pumps; and (6) mouse cages. The air from the chamber is taken at a port and, after passing through the soda lime and dryer–cooler, is reintroduced into the chamber at a distant port to create air circulation that homogenizes gas concentration in the chamber.

The specific device we implemented (Figure 2) was designed to expose up to 30 adult mice to chronic normobaric hypoxia. The hypoxic chamber made with transparent methacrylate walls (4 mm width; dimensions 50 × 61 × 84 cm) allowed 6 conventional mice cages (17 × 20 × 39 cm) for 5 animals each to be easily accommodated. The design of the setting was based on the analysis of the physiological variables corresponding to the demanding conditions of 30 mice with the highest possible adult weight of 40 g each, as explained in the following subsections. The dimensions and specifications of the different components can be modified as required if changing the number of mice or the animal species (e.g., rats or guinea pigs).

## 3. Results

### 3.1. Operation Procedure

Starting application of chronic hypoxia to mice requires the following initial actions: connecting the device to an electrical power line (preferably with backup), placing a recipient at the outlet tube of the Peltier-based air conditioning unit to collect the condensed water, filling the soda lime containers, connecting the device N_2_ inlet to a pressured source (e.g., a cylinder), setting its low-pressure/flow regulator to provide a N_2_ flow able to reduce the chamber O_2_ concentration from room air (21%) to the 11% set point in 10–20 min, placing the animal cages in the chamber, carefully closing the windows and pressing the power-on button.

During application of continuous hypoxia, the real-time values of O_2_ and CO_2_ concentrations, temperature and relative humidity within the chamber can be seen in the front panel of the control unit. The screen in the front panel shows an updated time course of O_2_ and CO_2_ concentrations for the last 48 h (e.g., allowing one to check that the system has worked correctly during an unattended weekend). The most important maintenance task is to replace the soda lime when required (either because it starts changing color from white to blue or because CO_2_ concentration starts to increase above desired values). When a mouse cage is extracted from the chamber (either for cleaning, feeding replacement or animal examination), the window must be closed immediately to minimize gas concentration changes in the chamber. Users should be aware of the information provided by the sensors’ technical data sheets regarding periodic calibration checking and sensor lifetime.

### 3.2. Device Performance

**Figure 3:**
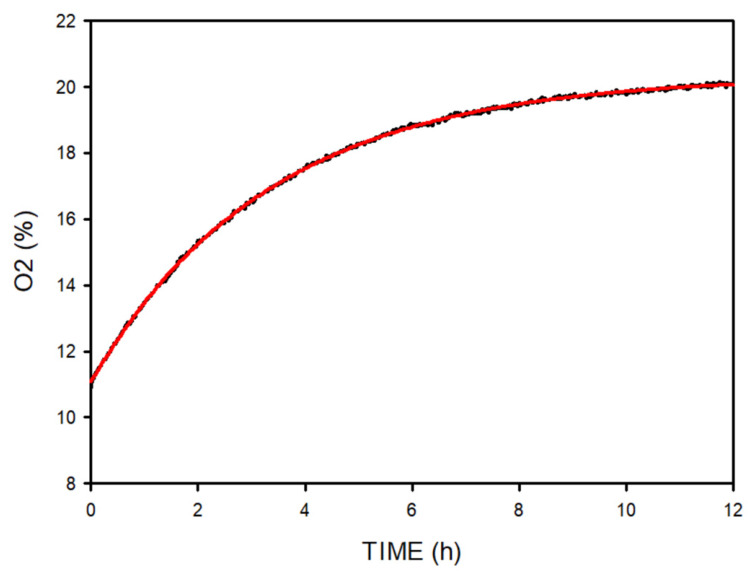
Time constant of gas exchange in the chamber. Passive change in chamber O_2_ concentration from stable 11% O_2_ after the N_2_ provision was interrupted at time zero. The black line is recorded data, and the red line is the exponential fitting with a time constant τ = 3.34 h (fitting R^2^ = 0.9996).

The passive dynamic change in gas concentration depends on both the chamber volume and the flow of gas leakage between the chamber and the room. We first characterized the passive time constant of gas concentration change in the chamber by measuring the O_2_ concentration when the provision of N_2_ was purposely interrupted when the chamber was at a stable 11% O_2_ concentration. Figure 3 shows an exponential variation corresponding to a time constant (τ) of 3.34 h.

**Figure 4:**
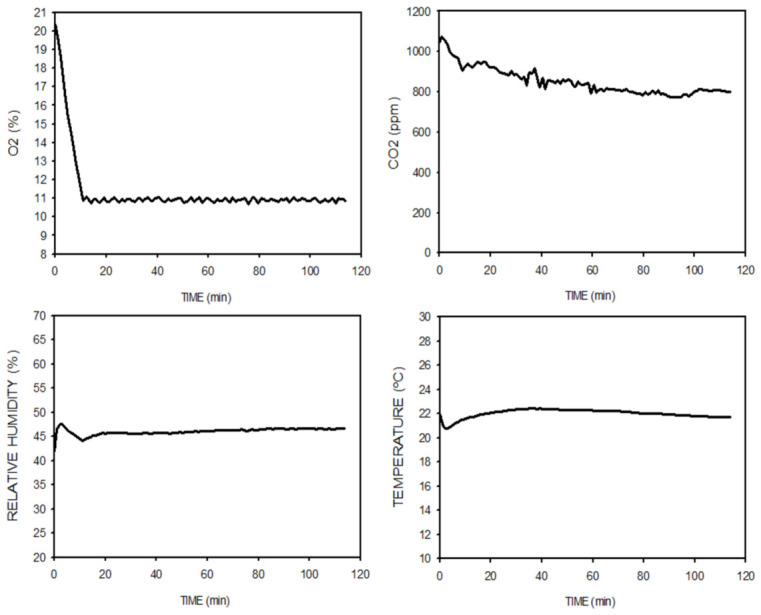
Example of the signals recorded inside the hypoxic chamber when, at time zero, mice were subjected to 11% chronic hypoxia from initial lab air conditions (20.5% O_2_, 1050 ppm CO_2_, 43% relative humidity and 22 °C).

The in vivo performance of the device was validated by applying it in research studies where mice were experiencing chronic hypoxia. Figure 4 shows an example of the O_2_ and CO_2_ concentrations and temperature and relative humidity signals recorded when 17 adult wild-type mice were subjected to 11% O_2_. The data in the figure starts when the mice were initially introduced into the hypoxia chamber under conventional lab ambient conditions and hypoxia application was initiated. As expected, O_2_ concentration decreased from lab conditions (21%) to the 11% set point and subsequently remained stable at this value with negligible oscillations (10.8–11.2%). CO_2_ absorption was very effective since its concentration was reduced from the initial room lab value ≈ 1000 ppm (i.e., 0.1%) to ≈800 ppm, demonstrating a considerable safety margin for maintaining CO_2_ levels within safe limits. The figure also shows that, after a very minor initial fluctuation resulting from the sudden N_2_ injection to lower O_2_ concentration until the set point, ambient temperature and relative humidity in the hypoxia chamber were maintained at values very close to the external lab room air, ≈45% and ≈22°C, respectively. N_2_ consumption to keep the 11–O_2_ state steady was only ≈2 L/min.

Uniform distribution of CO_2_ concentration within the chamber was confirmed by measuring it when 19 mice were maintained under steady-state conditions. Indeed, by placing a CO_2_ sensor at the nine different chamber sites potentially occupied by the mouse cages, we observed that the variability (coefficient of variation) in the CO_2_ concentration within the chamber was only 6% (see data in file “Supplementary results” in Supplementary Materials).

The maneuver consisting of opening a box door, taking out a mouse cage and closing the door again (e.g., for cleaning and checking the animals) minimally disturbs the O_2_ concentration within the box. Indeed, data from repeated measurements when the box was at a stable 11% O_2_ concentration with the 17 mice inside resulted in a maximum O_2_ concentration increase to 11.8% ± 0.1% (mean ± SD) 65 ± 9 s after ending the maneuver, and O_2_ concentration automatically recovered the 11% steady-state value 67 ± 12 s after the transient maximum increase (see recording example in file “Supplementary results” in Supplementary Materials). Thus, O_2_ concentration was altered by less than 1% for just 2 min.

The device allows application of long-term chronic hypoxia automatically (e.g., for weeks) provided that the N_2_ source remains available and that the soda lime is renewed as required.

These validation data and the suitability of the corresponding setting parameters correspond to the specific device built and animals employed. Users should adapt the device parameters, and validate them, in case of different applications, mainly depending on the number of animals and their metabolic rate (i.e., O_2_ consumption). It is noteworthy that once the device is designed for a given application (species and number of animals), the automatic control system based on gas sensors and gas source actuators (Figure 1) adapts its functioning to automatically account for the changes in metabolic rate of the animals (e.g., those caused by hypoxia, circadian cycle, physical activity, normal body weight increase, or fever in case of experimentally induced inflammation). To set the device parameters for a given application, the expected balances of O_2_, CO_2_, water vapor and heat can be estimated as indicated in the following paragraphs (Equations (1)–(6)), where computations correspond to 30 mice (40 g each).

**Oxygen balance**. The typical rate of O_2_ consumption in a mouse is 0.06 mL·(min·g)^−1^ [11]. Therefore, the total oxygen flow consumed (V′_O2_) by the 30 mice is ≈72 mL·min^−1^. The value of the oxygen fraction (Fi_O2_) in the hypoxic chamber that the user sets on the front panel of the control unit is achieved by mixing the flow of room air (V′_air_) and the N_2_ flow (V′_N2_) entering from the N_2_ source. The O_2_ flow entering the chamber is the amount introduced by the air pump (21%·V′_air_). The total flow of O_2_ leaving the chamber is the addition of metabolic consumption (V′_O2_) and the O_2_ contained in the total gas leaving the chamber (V′_N2_ + V′_air_), which is at Fi_O2_. Thus,

0.21·V′_air_ = V′_O2_ + Fi_O2_·(V′_air_ + V′_N2_), (1)

and hence

V′_air_ = (V′_O2_ + V′_N2_·Fi_O2_)/(0.21 − Fi_O2_) (2)

After an initial injection of N_2_ to achieve the desired level of hypoxia, e.g., a target Fi_O2_ = 0.11, minimization of further N_2_ consumption (V′_N2_ ≈ 0) during steady-state hypoxia would require V′_air_ = 720 mL·min^−1^ for V′_O2_ = 72 mL·min^−1^. This Fi_O2_ value is, in practice, ensured by using the O_2_ sensor signal to control the power of the pump injecting the airflow V′_air_ into the chamber.

**CO_2_ balance**. A critical condition to be achieved inside the hypoxic chamber is normocapnia, since the setting would tend to induce hypercapnia through the potential accumulation of the CO_2_ produced by mouse metabolism. Assuming that the ratio between CO_2_ production and O_2_ consumption, commonly known as respiratory exchange rate (RER), is equal to 1 in mice [11], the total flow of CO_2_ metabolically produced (V′_CO2_) is ≈72 mL·min^−1^. In case the CO_2_ fraction in the chamber (Fi_CO2_) is simply the result of the washout induced by the total circulating air (V′_air_ + V′_N2_), i.e., no CO_2_ absorption by soda lime, the CO_2_ entering the chamber by mouse metabolism would be balanced by the CO_2_ leaving the chamber. Under a steady state with minimal N_2_ supply (V′_N2_ ≈ 0, V′_air_ = 720 mL·min^−1^), CO_2_ washout would be negligible. Accordingly, the only effective way to reduce CO_2_ accumulation in the chamber is by continuously pumping a flow (V′_sl_) of the chamber air through a soda lime canister acting as a CO_2_ absorber. Then, the CO_2_ metabolically produced by the 30 mice (V′_CO2_ = 72 mL·min^−1^) is balanced by the CO_2_ absorbed by the soda lime (V′_sl_·Fi_CO2_) and the negligible amount of CO_2_ leaving the chamber (V′_air_·Fi_CO2_):

V′_CO2_ = V′_sl_·Fi_CO2_ + V′_air_·Fi_CO2_ (3)

Hence, for a pump flow V′_sl_= 60 L·min^−1^, the increase in Fi_CO2_ would be only ≈72 mL·min^−1^/60000 mL·min^−1^ = 12 · 10^−4^ or 1200 ppm (0.12%) above the typical Fi_CO2_ of room air, hence a safe value [12]. Since 1 kg of soda lime can absorb up to 260 L of CO_2_ [13], the soda lime required to drain the 72 mL·min^−1^ of CO_2_ production is 520 g·day^−1^.

**Water vapor balance**. Typical minute ventilation in mice is 1.46 mL·g^−1^ [14]; thus, for 30 mice, the total respiratory minute volume (V′_min_) is ≈1752 mL·min^−1^. Water vapor is released by mouse metabolism, mainly through breathing, thus tending to increase the relative humidity (RH) in the chamber air. The water content (V′_H2O_) in a flow V′_min_ of air at atmospheric pressure P_atm_, temperature T and RH is

V′_H2O_ = V′_min_·[P_H2O,sat_(T)/P_atm_]·RH_b_ (4)

where P_H2O,sat_(T) is the partial pressure of saturated water at temperature T. Accordingly, expiratory air (at 37 °C and water vapor saturated) and inspiratory air (at chamber temperature and RH_b_) contain different amounts of water vapor. Taking into account that the P_H2O,sat_ at 37 °C is 47 mmHg, for V′_min_ = 1752 mL·min^−1^, the water vapor content in the exhaled air (V′_H2O_) is 1752 mL·min^−1^·(47/760) = 108.3 mL·min^−1^. As P_H2O,sat_(25 °C) = 23.8 mmHg, V′_H2O_ in the inhaled air is 1752 mL·min^−1^·(23.8/760)·RH_b_ = 54.9·RH_b_ mL·min^−1^. Hence, the net water vapor produced by breathing is the difference content in expired and inspired air (108.3 − 54.9·RH_b_) mL·min^−1^. Thus, to keep RH_b_ at a reasonable value of 60%, a common value in lab animal facilities, the water vapor flow to be eliminated is 75.4 mL·min^−1^. Adding the water vapor released by mouse transepithelial evaporation (which amounts to ≈50% of water vapor loss by breathing [15]), ≈113 mL·min^−1^ of water vapor should be eliminated by the air dryer to avoid excessive humidification of the hypoxic chamber air. This amount of water vapor is eliminated by condensation in the cool dryer, specifically by cooling the airflow (V′_sl_ = 60,000 L·min^−1^) that is already circulated through soda lime to maintain normocapnia. Indeed, if a flow (V′_sl_) of chamber air (RH_b_ = 60%, 25 °C, thus containing 1128 mL/min of water vapor) is circulated through a refrigerated element that cools the air to a temperature (T_c_), the maximum content of water vapor in the refrigerated air will be reduced and thus the excess amount will condensate on the inner walls of the cooler. The maximum flow of water vapor contained by saturated cooled airflow V′_sl_ at T_c_ is 60,000 mL·min^−1^·P_H2O,sat_(T_c_)/760 = 79 mL·min^−1^·P_H2O,sat_(T_c_). Hence, the liquid water condensed by cooling V′_sl_ from 25 °C to T_c_ is 1128 − 79·P_H2O,sat_(T_c_). Accordingly, to eliminate the ≈113 mL/min of metabolically produced water vapor, it is required that P_H2O,sat_(T_c_) = 12.8 mmHg, corresponding to T_c_ ≈ 15 °C. Hence, a relatively mild ≈ 10 °C refrigeration from 25 °C would be enough.

**Heat transfer balance**. The metabolic heat dissipation by 25 g mice at common ambient temperature (20–25 °C) is 0.5 Kcal·h^−1^ [11], and thus the heat dissipated by the 30 mice (40 g each) would be (Q′_met_) ≈ 30 W. The net heat balance (Q′) in the hypoxic chamber is determined by metabolic production (Q′_met_), the heat dissipated by the soda lime in the absorption process of CO_2_ (Q′_abs_), and the negative heat transfer required for heating the previously cooled airflow V′_sl_ entering the chamber (Q′_sl_):

Q′ = Q′_met_ + Q′_abs_ − Q′_sl_ (5)

Specifically, Q′_met_ = 30 W, Q′_abs_ is 13.7 kcal/mol_CO2_ [13], which in this setting amounts to Q′_abs_ = 2.8 W (for CO_2_ density 1.8 g·L^−1^ at 20 °C), and Q′_sl_ for heating the airflow V′_sl_ from 15 °C to 25 °C amounts to −12.0 W (as computed for air density 1.2 g·L^−1^ and specific heat capacity 1 J·g^−1^·K^−1^). Hence, the net heat balance is Q′ ≈ 21 W. Taking into account that the chamber (50 × 61 × 84 cm) has a surface (A) of 2.47 m^2^, that the chamber walls are made of 4 mm width (d) transparent polymethyl methacrylate (thermal conductivity: K = 0.18 W·m^−1^·K^−1^), and that the basic equation for heat transfer is

Q′ = K·A·ΔT/d(6)

it is determined that passive dissipation of Q′ = 21 W through the chamber walls would be achieved for a ΔT ≤ 0.2 °C. Hence, heat transfer balance is achieved for a hypoxic chamber temperature that minimally differs from room temperature.

For simplicity and taking into account that the process of reducing O_2_ from room air (21%) to the chronic hypoxic target (11%) only occurs once at the beginning of the experiment, we did not include an automatically controlled time course for the O_2_ reduction. This initial process of animal adaptation should be carried out under the direct supervision of the investigator, and they can manually modulate the rate of hypoxia application by partially opening one of the box doors. However, it would be easy to include a few sentences in the code to limit the rate of O_2_ concentration reduction.

## 4. Discussion

In this work, we have quantified the O_2_ concentration by its percentage in air, as is it more normal when assuming operation at sea level. However, in case the chamber is used in high-altitude places where atmospheric pressure is below that of sea level, it should be taken into consideration that the physiologically relevant variable for O_2_ concentration is its partial pressure.

The experimental setting presented herein may be useful for facilitating the study of pathophysiological mechanisms and potential therapies in diseases where chronic hypoxia plays a significant role [16]. Hypoxic challenge is present in a variety of conditions. The most prevalent are those related to diseases that cause deficient tissue oxygenation, whether due to respiratory failure (e.g., chronic obstructive pulmonary disease [17], asthma [18], fibrosis [19], pulmonary hypertension [20]) or cardiovascular dysfunction (e.g., heart failure [21], coronary artery disease [22], congenital heart defects [23]). Chronic hypoxia can also be relevant to the fetus in gestational diseases because of either placental abnormalities or maternal conditions such as heart/lung disease, anemia, preeclampsia, diabetes, or sleep apnea [24]. Furthermore, hypoxia is a well-known condition experienced in cancer due to abnormalities in the vessels within rapid-growing tumors [25], inducing aggressive cell adaptation, thereby promoting proliferation, metastasis, and resistance to chemotherapy and radiation therapy [26,27]. Also relevant are the negative health consequences of living at high altitudes, where the partial pressure of atmospheric oxygen is severely reduced, thus inducing tissue hypoxia [28], increasing risk of pulmonary hypertension [29], potential cognitive impairment [30], kidney problems [31] or male [32] and female [33] fertility reduction.

The use of animal models of chronic hypoxia allows us to study its deleterious effect at the cell, tissue, organ and systemic levels. In particular, such research provides clues on the inflammation and oxidative stress triggered by hypoxia by altering mitochondrial function and enhancing excessive production of reactive oxygen species [34,35,36].

From an engineering perspective, the solutions presented herein represent an advance in the field. Open-source solutions based on microcontrollers are available [37], but they are more expensive and require a continuous N_2_ flow [9], thus increasing the total experimental costs. Moreover, the device developed in this work incorporates the security mechanism required by ethical committees for animal experimentation.

It is important to emphasize that the aim of this work is not to introduce a standardized, commercially available “plug-and-play” device. Instead, the manuscript presents a straightforward methodology that enables researchers to construct an inexpensive and easy-to-fabricate chronic hypoxia system, particularly suitable for laboratories with limited access to nitrogen supplies. A key feature of this open-source approach is that system performance will depend on the specific dimensions, materials, O_2_ and CO_2_ sensors and construction details implemented in each laboratory. Therefore, any device built following the procedure described herein should be validated under its intended experimental conditions, particularly with respect to the target animal species and the number of animals housed. Given the inherently customizable nature of the proposed system, a direct comparison with commercially available hypoxia devices was not undertaken. Such comparisons would be difficult to interpret, as performance is expected to vary depending on the specific implementation. Rather than proposing a fixed device with predefined specifications, the goal of this work is to provide an open and accessible methodology that can be adapted to the needs and resources of individual laboratories.

## 5. Conclusions

An easy-to-build setting for automatically subjecting rodents to chronic hypoxia in facilities with minimal nitrogen supply has been designed and tested. All the detailed technical information to build the device (e.g., electronic circuits, control microprocessor, 3D-printed components) is open-source-provided (Supplementary Materials) so that any interested reader can reproduce or adapt it for their application. Since it does not require a high-flow N_2_ source, this setting can be useful for expanding animal hypoxia research in premises where conventional commercially available devices are not usable.

## Data Availability

All detailed data are provided in this main text and in the Supplementary Materials.

## Supplementary Materials

The following supporting information can be downloaded at: https://www.mdpi.com/article/10.3390/life16071140/s1.
