# Supplementary material for: Optimized Open-Source Setting for Subjecting Rodents to Chronic Normobaric Hypoxia in Facilities with Minimal Nitrogen Supply
Source: Life (Basel). 2026 Jul 9;16(7):1140. doi: 10.3390/life16071140 (PMC13412489; doi:10.3390/life16071140)
Supplement: Supplementary file 1 [file life-16-01140-s001.zip › Buiding technical instructions.pdf]

1. *Design files*

All the design and software files necessary to build the device presented in this work are distributed under the GPL v3 license and they can be found in the supplementary materials of this manuscript at the following public repositories https://data.mendeley.com/datasets/t7dk933sjm/1

2. Bill of materials

**Component**	**Quantity**	**Cost per unity €**	**Total Cost currency €**	**Source of materials**
Sensor O_2	*1*	62,30	62,30	https://es.farnell.com/dfrobot/sen0322/i2c-oxygen-sensor-module-arduino/dp/3879708?gad_source=1&CMP=KNC-GES-GEN-SHOPPING-Pmax-Catch-all-05-Dec-23&gross_price=true
Sensor CO2, temperature and relative humidity	1	61,14	61,14	https://es.farnell.com/seeed-studio/101020952/m-dulo-sensor-arduino-raspberry/dp/4007751?st=modulo%20sensor%20%20co2
Voltage regulator 5V and 9V	2	0,28	0,56	https://www.amazon.com/valores-Paquete-regulador-positivo-corriente/dp/B07T5ZHY63/ref=sr_1_1_sspa?__mk_es_US=%C3%85M%C3%85%C5%BD%C3%95%C3%91&crid=1651XJCJIY90X&dib=eyJ2IjoiMSJ9.z7VZ01yDMzS7FNoFVZYjfIIDIqJf7MWuKacqC0FVcexkbaaq2K3koWWNRLSjCUnNocgOZhSxSL_K2NLBBZrMZcpmaEX61JZhaHNEK6GLg-pEYKA2nXRwSKnqndWUS3hKDuTBenbCbF9ouzxqIJhS3vUE_hhOFrJp8Tlc9ngNTITlqc6fLt1BAs_ijswPUxupFI1jSGK1lUobc-yQ_mRvKIzIJFYo4byvBh0iwoiQtwk.e5isTzn0mwmTiPh46biH3ZfvGoMPDzyBZDeiYAUNsKQ&dib_tag=se&keywords=voltage+regulator+7809&qid=1749637707&sprefix=voltage+regulator+780%2Caps%2C158&sr=8-1-spons&sp_csd=d2lkZ2V0TmFtZT1zcF9hdGY&psc=1
Diode	1	0,04	0,04	https://www.amazon.com/conmutaci%C3%B3n-miliamp-voltios-silicio-electr%C3%B3nicos/dp/B07Q4F3Y5W/ref=sr_1_1_sspa?crid=19S08TO69SSPJ&dib=eyJ2IjoiMSJ9.G4ZHuurkn3IVHsyuKzuoxxIKGXN42qvZAc9mcrW5r69d231gJdkC9262W6Y9Ge7VLNqRy653RUZFWEvndlAY5xd59nQGcFeStKJK1_vYzKzwS6160cq9R0J4r7gITSLqXn6BMuL67mi8kVQTMqTT2NHGUpL0ptQOIsJUrpLD2EnGLzXHuZAQ9FXGqVdy_-a-TAHyH_NyhFEjnBrFpiu8voqXx_8EnP7By8SCqog0cLuR0ymxSKV6cAbdcwyaRp9ebK8oHe41a7807tuTpKDn0sVd7oFUAog0Dy4GwfcQmjw.AWgWGJvW0Wjxyzk9oo0Ga_ChksybQGJP_y22Lfu2cvI&dib_tag=se&keywords=in4148&qid=1749637823&sprefix=%2Caps%2C115&sr=8-1-spons&sp_csd=d2lkZ2V0TmFtZT1zcF9hdGY&psc=1
Transistor	1	0,28	0,28	https://www.amazon.com/valores-transistor-potencia-epitaxial-Darlington/dp/B08BFYVK6C/ref=sr_1_2_sspa?__mk_es_US=%C3%85M%C3%85%C5%BD%C3%95%C3%91&crid=F7L9BPIUJJ1J&dib=eyJ2IjoiMSJ9.h3c-xIy3MyTNjNTX3uCVtQcwfWZK_sKfZ5uAxLmZxsqRtPa0gU6bxTYAyV5DnWuyAXByYs1n1nX0i6Q_I5MTe88pNAxBZxrPfEN8voxaQCtiZdJVnQwGgVRG-ODWzeXTkUsDoi6EbqcQMcgAnbQ2VqfPOWZgFRMchaxT0K9n-Gp6LGWk1V-iedqbZIP-o_bcyz1OLHvcxVl1vjiv74yYgciAfzE5yGDDIyRHC4qiHCw.mcSg2LgzvVTPouRlbtI4MDXSFdDEva1NHrZGLCBTpZU&dib_tag=se&keywords=tip122&qid=1749637875&sprefix=tip12%2Caps%2C198&sr=8-2-spons&sp_csd=d2lkZ2V0TmFtZT1zcF9hdGY&psc=1
Solid state Relay	1	2,20	2,20	https://www.amazon.com/-/es/HiLetgo-m%C3%B3dulo-estado-Control-fusible/dp/B00WSN9CJC/ref=sr_1_6?__mk_es_US=%C3%85M%C3%85%C5%BD%C3%95%C3%91&crid=1DCIOST74VCPI&dib=eyJ2IjoiMSJ9.q91PYm6OKAKVxQl4ebBf0AcV_wFfLUw6yFpImY74y9NNuAwuMA_hpIqRP2tM66p4flq29UABM9fA-Vt8GxLx1_6p2ie44_AJ84mtp5V95vCcb2_E7tkDR2bruMwsij8DGnkhkGYR3yGb3K202Yot5Uw3FOzQ5Em4ew4ZXLH8U5mn9tf4yMoJdQZ_yI1NPchNJZzmKsWA_Omo89YqgdAJFDpTr6nQ7RvzFNO0pdqxyZg.B-yP_CUBkbA0UKhlF3XE1GJNXnzZo4iaBUsoYxd5gl0&dib_tag=se&keywords=rele+solid+state+5V&qid=1749637984&sprefix=rele+solid+state+5v%2Caps%2C131&sr=8-6
Connectors	6	0,11	0,66	https://www.amazon.com/s?k=conectores+arduino&__mk_es_US=%C3%85M%C3%85%C5%BD%C3%95%C3%91&crid=ZOJJGKBHPROE&sprefix=conectores+arduino%2Caps%2C133&ref=nb_sb_noss
Capacitors	4	0,04	0,16	https://www.amazon.com/ALLECIN-surtido-condensadores-electrol%C3%ADticos-aluminio/dp/B0C1VBXCQM/ref=sr_1_1_sspa?__mk_es_US=%C3%85M%C3%85%C5%BD%C3%95%C3%91&crid=O5JYUB0HNKY2&dib=eyJ2IjoiMSJ9.ekYH7jx2EeGYfnpDMkTUbA5_8nAZM5NXRF7ISTCDYRdiGRRate4MlrQd9ldvZs-A_pzD-zjApyYWkhjoUOqNALpVcJsc6DD3JHlReRoBcxMOy5HnZgoPPDGKG

				Q7eKlroLYErlCkXUuGwYEHo_DbuP4dZ3WHupx5R5NgErb4ax20-0bVA56GPbf6AHFW0x2BVjzMCRqJcM72pPARxXlipVzthtF2eEvVnVx2oaxKJv7E.rax_WABdj5Kg3iZg0-DCwqXoqhX58FBhrXxqFDcFrrk&dib_tag=se&keywords=condensadores&qid=1749638632&sprefix=condensadore%2Caps%2C169&sr=8-1-spons&sp_csd=d2lkZ2V0TmFtZT1zcF9hdGY&psc=1
Arduino Mega	1	23,25	23,25	https://www.amazon.es/ELEGOO-Microcontrolador-ATmega2560-ATmega16U2-Compatible/dp/B06Y3ZHPWC/ref=sr_1_2_sspa?__mk_es_ES=%C3%85M%C3%85%C5%BD%C3%95%C3%91&crid=16IXP8IYVEZIP&dib=eyJ2IjoiMSJ9.VNlP3CQ0NzQDPWLu2uC7KkHA__lwuDLf45_9fQSmtbWX2NOfw9qL2kbiUGF2MnDCMVOVg4_-bUWaN20Oo-pyASW_fsBa_0BKxKMcKOIPvQIcAQr6LkANQDjtDT_nyyHx2ukyr-PD484nwxI2IANpNtl_E_-62_pEWMiufkU8QHI7q1S8JpvgGJMgdeHwqG-1ufXxIfMmGLtVCMEVGeAfOb1MSvMF3AFAV1wK7aIIlRbpWm8kf6G-NnivmR22WzIv0Qw8t_W8ghw9jDU8I6Lsm2-z2Id8eIaxHEbcPxL0E-4.c8KUwVEiJ7T0gRfOboB311pEUyYhsvdQTwCAj5wkmN4&dib_tag=se&keywords=arduino+mega+con+pantalla&qid=1749642004&sprefix=arduino+mega+con+pantalla%2Caps%2C90&sr=8-2-spons&sp_csd=d2lkZ2V0TmFtZT1zcF9hdGY&psc=1
Screen 3,5’’	1	18,99	18,99	https://www.amazon.es/Binghe-Bol%C3%ADgrafo-Contacto-Resoluci%C3%B3n-Compatible/dp/B0D6B9M4ZH/ref=sr_1_4?__mk_es_ES=%C3%85M%C3%85%C5%BD%C3%95%C3%91&crid=16IXP8IYVEZIP&dib=eyJ2IjoiMSJ9.VNlP3CQ0NzQDPWLu2uC7KkHA__lwuDLf45_9fQSmtbWX2NOfw9qL2kbiUGF2MnDCMVOVg4_-bUWaN20Oo-pyASW_fsBa_0BKxKMcKOIPvQIcAQr6LkANQDjtDT_nyyHx2ukyr-PD484nwxI2IANpNtl_E_-62_pEWMiufkU8QHI7q1S8JpvgGJMgdeHwqG-1ufXxIfMmGLtVCMEVGeAfOb1MSvMF3AFAV1wK7aIIlRbpWm8kf6G-NnivmR22WzIv0Qw8t_W8ghw9jDU8I6Lsm2-z2Id8eIaxHEbcPxL0E-4.c8KUwVEiJ7T0gRfOboB311pEUyYhsvdQTwCAj5wkmN4&dib_tag=se&keywords=arduino+mega+con+pantalla&qid=1749642106&sprefix=arduino+mega+con+pantalla%2Caps%2C90&sr=8-4
Chamber polymethyl methacrylate	2,5m2	65,72(m2)	164,30	https://planchasdeplastico.es/producto/policarbonato-transparente-3-mm/?gad_source=1&gad_campaignid=16996510449&gbraid=0AAAAAoVwwA-2JSUGanHN_CR0-AZc1lvjj&gclid=Cj0KCQjw0qTCBhCmARIsAAj8C4Y5dcppkkNshgAS0GoqejuNiEiCO95D5VttoEFx4nHkqewEDAKf7SoaAnJJEALw_wcB
Rack	3	11,00	33,00	https://www.amazon.es/Mallard-Ferri%C3%A8re-cromado-rejilla-60/dp/B00BMKWP5U/ref=sr_1_7?__mk_es_ES=%C3%85M%C3%85%C5%BD%C3%95%C3%91&crid=2V6WL13VKL0ZY&dib=eyJ2IjoiMSJ9.D1aLnzF4G36EJHClT1wbHuzBq6CHue7ZO_5WTr09lPf_FcX3ORDatQU4OY5709laVOA0idmW5NpqLC6RQz7T87oYfxxrDxjSR5eTrB0yLd1bjZbrDPnmBd7xqRa9mAxg3pcdKfTQlQGtVIfal4jVlV9tNLabpyse-1In13VbL5By94Qt2BkDkJo4coagITwtE3pNG-Drcpuch8YyzA4YqWY2huacB4vj4O4oZJ7au3IjG-Y1rjsIMG4AVEJtxHMyniUypJ0bWs_USfhGiPeVA_JXeFG7aufLo6DtSsklo-c.9nLvIyT4-DDJM9Y6EGfGY1Slq_SyAkWfgoQLRpgNxXE&dib_tag=se&keywords=rejilla+60x50&qid=1749639549&sprefix=rejilla+60x50%2Caps%2C83&sr=8-7
Pump 30l/min	1	61,18	61,18	https://www.amazon.es/Hailea-bomba-minuto-incluye-hidrop%C3%B3nico/dp/B00NSOQZO0/ref=sr_1_6?__mk_es_ES=%C3%85M%C3%85%C5%BD%C3%95%C3%91&crid=2QV3ZCZZQW2EP&dib=eyJ2IjoiMSJ9.8EvrXsj1Rve6ppqRPADz0-VaZ0Vpe3Ad9jM2YO1LJHfGVrsnl4xbu3vmb0839HdqjEGE8U7JU9FCOhZENWH55rtt5RVu7eWzqa-n7OEE70Ot9CG2JJupy_W4fQCEEgj1ojhxiMCgqONskinhanDqchDC4PTigzlI2OZqpo_y1HUKPAySq8f8n4WtXCsYTqWiHs4TX7fCgu7U6xTNvHZS8mB-U7dgI-JvHv9Z9ODIOE6h5fac7dldxlGhVDIBLOF2mTGdx8rXOzodLoEyiE1r_xtG-xHwmzzBHrQsUgGERRc.QgNQ_J8WkHaYqVuGzuyWAp5TwTee0tubDkAzO8SgjEs&dib_tag=se&keywords=bomba+60l%2Fmin&qid=1749639830&sprefix=bomba+60l%2Fmin%2Caps%2C94&sr=8-6
Pump 60l/min	1	110,00	110,00	https://www.amazon.es/AquaForte-aluminio-Silenciosa-Capacidad-regulable/dp/B006SYHCI0/ref=sr_1_5?__mk_es_ES=%C3%85M%C3%85%C5%BD%C3%95%C3%91&crid=1Z9OHLAOKFU5L&dib=eyJ2IjoiMSJ9.NRkweEtIZU8--IlxdeJnBVnA57aO3bZv5hAEsbZEYcl9xBXcXFNMVAeKlU_86n4GJzWpJ4OIB5EVtu1BudyX06OpzDGO0m-QAcaWxxlnw97CIZDvWj0Q6BCLHxdqOjyrizC-5bgqUfTjGLc7AgJx9N7xc5UT6tWYHqJTycqoAMjZkBHAgosIT_-LUFZez5Z_CAMOPIR3IbjEVCJU9N_bvnvFdB68xiLwvSfY2hTUrhiUBE75D232bex3DA3G01LUZhVONH23IfRAz2U_0am5jKcT0EFB2WKabKOpAJOVNUM.SmnSqG7K3XQG0_bTTbTVbFH7hzXOCEkY8uRXlZar1lc&dib_tag=se&keywords=Bomba%2Bv30&qid=1749640500&sprefix=bomba%2Bv30%2Caps%2C130&sr=8-5&th=1
3D printing	-	31,00	31,00	https://es.farnell.com/ultimaker/1609/filament-pla-black-750g/dp/2992628?gross_price=true&CMP=KNC-GES-GEN-SHOPPING-Pmax-High_ROAS&gad_source=1&gad_campaignid=18071281895&gbraid=0AAAAAD8yeHlKxiGMy79WCfJwNBT_BsqP9&gclid=Cj0KCQjw0qTCBhCmARIsAAj8C4b-IsF7T5oluiyewkZV-nNr8tDFVX0Vy5St76HWuHN5j-02Z0rQICEaAhJBEALw_wcB

Thermoelectric cooler	1	47,41	47,41	https://www.amazon.es/Refrigerador-Refrigeraci%C3%B3n-Termoel%C3%A9ctrico-Enfriamiento-Semiconductores/dp/B0F6NWNWJM/ref=sr_1_16?__mk_es_ES=%C3%85M%C3%85%C5%BD%C3%95%C3%91&crid=1XPI9W7G6FJMY&dib=eyJ2IjoiMSJ9.9IY2M3EO0bKYIISgJaOjbFR0CO1MNnuPKF7FQmBmf24793Shg3oHhO-N_JNhPTuMwtWic163ggGxBsgEaRs6K16S-PeHerDs4niKZfdYLnI9DrVWBIBR1iV60ukXPu3hhjJTvsIpVfQ1XD-gxILKkw2GAAQosMOAUKuB3SloCD-4lxuFh2BLEdseAl5S91JsiijAaGn6LtfQBiIioHr3EaD2cnSsogdszxBCcni6g-tGlKIw5CnGXptQwrq8BEX4UCFTLGcdRjignZ0kLzgelIU7xlUn5pjFYNRXYykJEW0.kDaw92Wqmkk6qb-3Jdq5KTzDpfY6PgVISbfYPW0V8aU&dib_tag=se&keywords=peltier+12v+120W+liquido&qid=1749724430&sprefix=peltier+12v+120w+liquido%2Caps%2C89&sr=8-16
Water cooling radiator	1	15,99	15,99	https://www.amazon.es/CENPEK-Radiador-refrigeraci%C3%B3n-intercambiador-computadora/dp/B09FXL787S/ref=sr_1_12?__mk_es_ES=%C3%85M%C3%85%C5%BD%C3%95%C3%91&crid=PQGXM2YHEEV3&dib=eyJ2IjoiMSJ9.yjbbkS_YR53lsFDVB9rz0eYd5sZpphUjuTHx0GHaUkYI5UlNnAXcYGrK5NPn7pJfDK7bzqOrTpZhn_NPIxIlyE2goUsz_bcNBKzDvYGkaIZlo9KPsHoiczEU67CN8_tpfdQOO35OF_DK9ZAaofcYhHLr3qrTsUEJqPhkwz7V0Bg8SO2REwV15OTBLg_uwcM5tloSxGjuGmAhrcS_X7Qrxw18W9LjGTun564uKLiel05dAQiT5TZ6nAGbaw8igOMGZmzpsWvEZrDNOEXwrxasGjp5itvNilLNFstgyNaFOJc.G1-K8oQzQAXi-uTpVrxW7Fyd0eNC0x3PsQHYMwMlq-4&dib_tag=se&keywords=radiador+liquido&qid=1749724514&sprefix=radiador+liquido%2Caps%2C77&sr=8-12
Water cooling pump	1	19,72	19,72	https://www.amazon.es/Diyeeni-refrigeraci%C3%B3n-expansi%C3%B3n-m%C3%A1x-Bomba-enfriamiento/dp/B07ZRQRV7J/ref=sr_1_4?__mk_es_ES=%C3%85M%C3%85%C5%BD%C3%95%C3%91&crid=2PGY6ZH64SWIS&dib=eyJ2IjoiMSJ9.fLA9IrZ-4xIiJiCZDNLIo0iOr0srTdzEtk26K6yuy7gC27QZgzKO3yCZpTJPTes98E8sfvWLDY58QdMIxf7WFjIln4dTfSQemugONaQwo8cqYc70UEWTb35qg50KeciM-G5VzBf4mZiaAk483FHoIRd8rsp9fGP9PY_fHRqzVkEE9MAFjzffeJJapV2YuOewTKz0QHwzmhtHavNzgWzUONq1C4nojCNiIV_by89kzD9YEq5_OL82FUaG-W6l5QxrdNt7rga4DEEUKZW26d28iO6IUbgxF22iBQ_jiEh9h6o.T2DoqO2ThfgHihFKC9f4I2e8n3kWlOl2-tvC-n82Zew&dib_tag=se&keywords=bomba+liquido+refrigeracion&qid=1749724631&sprefix=bomba+liquido+refrigeracion%2Caps%2C77&sr=8-4

The total cost of the materials for building the device is **633,77€**. Materials such as resistors, LEDs, pin connectors, capacitors, PCB, ICs, and fuse holders were purchased as a kit, however, not all materials available in the set were used when building a single device. Most of them can be also easily reused from obsolete/damaged consumer electronic devices or household appliances.

3. *3D Design and Printing*

The chamber was built using polymethylmethacrylate sheets, forming a sealed enclosure with three hinged doors that allow the insertion of mice cages. To ensure proper closure, a metal rod is used to apply pressure on each door against the chamber frame. Custom 3D-printed holders were designed and fabricated to hold the metal rods securely in place. On the side of the chamber, additional 3D-printed holders are used as storage for the metal rods when they are not in use. Beyond structural mounting, 3D printing was also leveraged to develop custom protective enclosures for the gas sensors affixed to the chamber. These cases were specifically designed with dedicated slots and openings for efficient gas exchange. A custom 3D-printed case was also created to house the electronic circuit incorporating the Arduino board. This enclosure includes ventilation slots to prevent overheating, a front opening for the display module that visualizes real-time data and system graphics, and a rear connector interface for power supply integration. 3D printing was also employed to create accessory components for the soda lime containers. These components ensure optimal airflow for efficient CO_2

removal. Furthermore, a funnel specifically designed for refilling the soda lime containers was also produced using a 3D printer. Figure 1 shows the 3D-printed components.

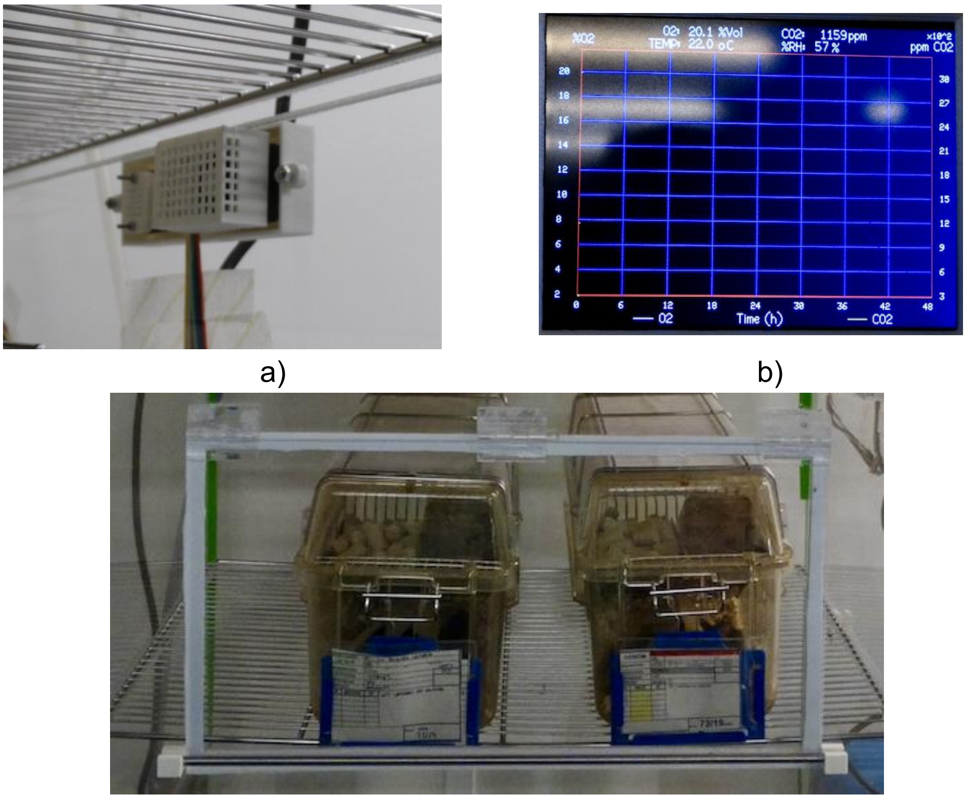

a) b)

c)

Figure 1: 3D-printed components, including the sensor enclosures (a), the Arduino and electronics housing (b), and the metal rod supports used for sealing the chamber doors (c).

4. ***Electronics***

The electronic system, shown in Figure 2, has been designed to interface the gas sensors with a microcontroller unit (MCU), specifically an Arduino Mega, while also enabling control of electromechanical actuators such as a solenoid valve and pumps via a transistor switch and a solid-state relay, respectively. An I²C-based Sensirion SCD4x sensor is employed for measuring CO_2 concentration, temperature, and humidity, while oxygen levels are monitored using a DFRobot I²C oxygen sensor module. The schematic is organized into distinct functional blocks: sensor interfacing, power regulation, and actuator control. The system operates from a single 12V DC power source. Two linear voltage (7805 for 5V and 7809 for 9V) regulators are used to derive the required supply voltages for the other components.

The injection of N_2 into the chamber is allowed by a solenoid valve driven by TIP122 NPN Darlington transistor that acts as a switch. A flyback diode (1N4148) is placed in parallel with the solenoid coil to protect the transistor from voltage spikes. This valve remains closed except when activated. The introduction of room air through the pump is controlled via a digitally-activated solid-state relay, which provides electrical isolation and long-term durability in switching operations.

To regulate humidity inside the chamber, a cooling system based on a Peltier cell is used. This system consists of a thermoelectric cooler, a radiator, and a fan. The Peltier cell cools the air circulating through the chamber. As the air cools, it condenses on the cold surfaces inside the dryer. This process effectively removes the water vapor produced by the mice, which helps maintain a stable relative humidity. The cooled and dehumidified air is then returned to the chamber.

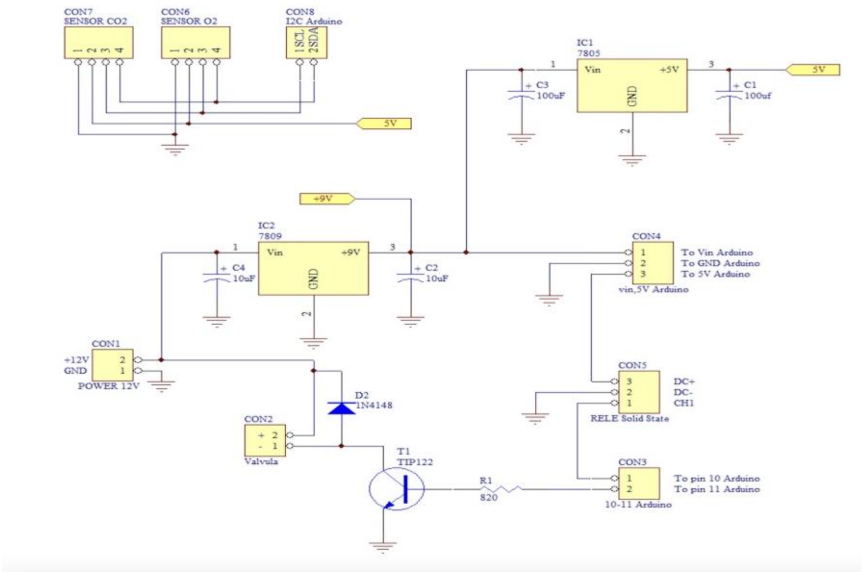

Figure 2: Schematic

5. *Arduino control*

The Arduino firmware is structured to manage real-time acquisition, display, control, and recording of oxygen (O_2) and carbon dioxide (CO_2) concentrations, along with temperature and relative humidity. These values are periodically sampled and visualized on a 3.5" TFT LCD driven by the TFT_HX8357 library. Upon initialization, communication with all peripherals is established via I²C and SPI interfaces. A graphical interface is then rendered on the display, including axes for time and gas concentration, grid lines, and fixed legends for the visual interpretation of data. A rectangular plotting area is drawn for graphing real-time measurements. Initial error checks are performed to verify sensor integrity. The main function of the code contains the core operational logic and it is executed iteratively. At each iteration, the system checks whether new data is available from the CO_2 sensor. In the event of a sensor error (basically checked by the I2C bus), the nitrogen solenoid valve is closed and the air pump is activated, so animals are subjected to room air until the error is fixed. Once valid data are retrieved, the CO_2 concentration, temperature, and humidity values are updated. Simultaneously, the O_2 sensor performs an averaged measurement based on ten samples, providing a smoothed value of the ambient oxygen concentration. The numerical values of humidity, temperature, CO_2, and O_2 are updated on the display interface every 3–5 seconds, in accordance with the conversion time of the CO_2 and O_2 sensors. Approximately every 7 minutes, the system plots instantaneous values of O_2 and CO_2 on a dynamic scrolling graph. The X-axis represents time (scaled to cover 48 hours with 412 data points), while the Y1 and Y2 axes are scaled to appropriately display O_2 (%) and CO_2 (ppm) concentrations, respectively. A circular buffer mechanism ensures efficient redrawing of the graph without overloading memory. Figure 3 shows the graphical interface on the

display. Simultaneously, the system logs environmental data to an SD card every 60 seconds, including timestamp, temperature, humidity, O_2 concentration, and CO_2 concentration. This allows for offline analysis and long-term monitoring.

A bang-bang controller is used to maintain the gas mixture at the desired values during the experiments. On the one hand, if the oxygen concentration exceeds the setpoint (11% in the examples), the system activates the N_2 valve until the level reaches the setpoint. Once this setpoint value is reached, the valve is deactivated, cutting off the N_2 supply. On the other hand, if the O_2 level drops below a critical value (setpoint-0.5% in the examples), or the CO_2 concentration exceeds a critical value (2000 ppm), the N_2 valve is automatically closed, and a room air (i.e., oxygenation) pump is activated until the O_2 / CO_2 levels are restored to the defined setpoints (11% and 1500 ppm respectively). Depending on the specific experiment aim, if the 2000 ppm threshold is considered too high, it can be lowered by simply modifying its value in the code.

The choice for bang-bang control is made, apart from being simple to implement, because this type of controller offers faster spin up times. This is especially important in applications where the system needs to be frequently manipulated (long periods with the door open or the equipment switched off). Nevertheless, it is well-known that bang-bang controllers present two main drawbacks, which could lead to experimental problems if very precise FiO2 is required: ripple in the signal and risk of overdamping. As is shown in the next section, we have not observed overdamping, and the ripple is within acceptable range for most applications. If more precision is required to mimic certain pathologies, we advise implementing a more complex control scheme, such as proportional-integral-derivative (PID). It should be noted that, however, the PID parameters are more difficult to tune, so the intervention of technical personnel will be needed whenever any of the experimental parameters are changed (number of animals, gas pressures, etc.)

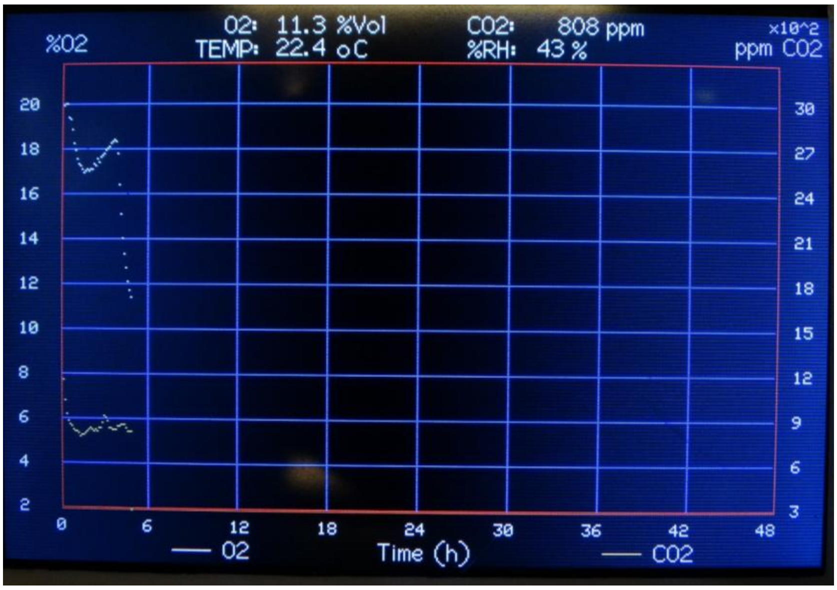

Figure 3: The graphical interface on the display. This example shows the first 5 h of a recording where the N_2 source functioning was intentionally altered to more clearly observe sudden changes in O_2 concentration.
