# Supplementary material for: Optimized Open-Source Setting for Subjecting Rodents to Chronic Normobaric Hypoxia in Facilities with Minimal Nitrogen Supply
Source: Life (Basel). 2026 Jul 9;16(7):1140. doi: 10.3390/life16071140 (PMC13412489; doi:10.3390/life16071140)
Supplement: Supplementary file 1 [file life-16-01140-s001.zip › FailSafe/FailSafe.pdf]

Fail-Safe Device

Hardware Description

The proposed device has been conceived as a secondary safety system. It is designed to ensure animal welfare within the experimental chamber, even in the event of power interruptions. Its primary function is the continuous monitoring of carbon dioxide (CO_2) and oxygen (O_2) concentrations, combined with the automatic activation of a ventilation pump whenever predefined threshold values are exceeded. In addition, an integrated alarm system alerts the operator when environmental parameters fall outside the acceptable range.

To guarantee continuous operation, the device is equipped with an uninterruptible power supply (UPS), thereby maintaining functionality during temporary power interruptions.

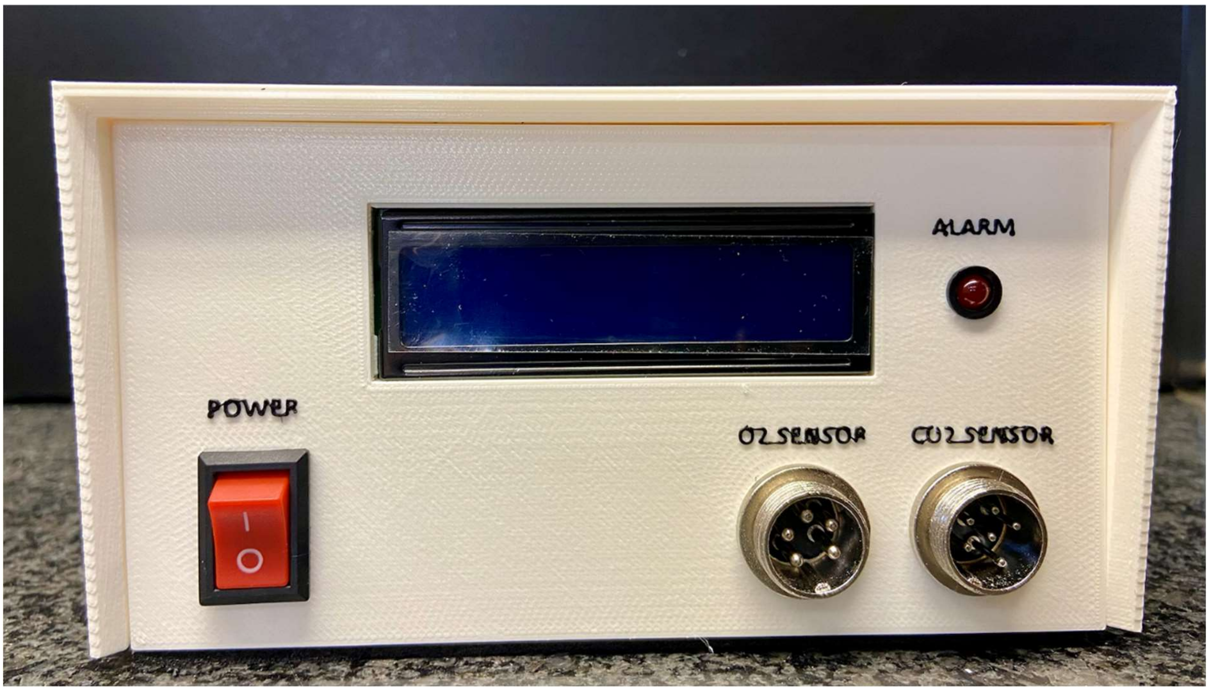

Fig 1. Front panel of the secondary safety system

1. Design files summary

All the design and software files necessary to build the device presented in this work are distributed under the GPL v3 license and they can be found in the supplementary materials of the manuscript at the following public repository: https://data.mendeley.com/datasets/t7dk933sjm/1

Table 1. Files summary

Design file name	File type	Open source license	Location of the file

Enclosures and lids	STL	GPL v3	FailSafe folder
Code	ino file	GPL v3	FailSafe folder
PCB Layout	pdf and jpg file	GPL v3	FailSafe folder

2. Bill of materials summary

**Component**	**Quantity**	**Cost per unity €**	**Total Cost currency €**	**Source of materials**
Sensor O_2	*1*	62,30	62,30	https://es.farnell.com/dfrobot/sen0322/i2c-oxygen-sensor-module-arduino/dp/3879708?gad_source=1&CMP=KNC-GES-GEN-SHOPPING-Pmax-Catch-all-05-Dec-23&gross_price=true
Sensor CO2, temperature and relative humidity	1	61,14	61,14	https://es.farnell.com/seeed-studio/101020952/m-dulo-sensor-arduino-raspberry/dp/4007751?st=modulo%20sensor%20%20co2
Voltage regulator 9V	1	0,28	0,28	https://www.amazon.com/valores-Paquete-regulador-positivo-corriente/dp/B07T5ZHY63/ref=sr_1_1_sspa?__mk_es_US=%C3%85M%C3%85%C5%BD%C3%95%C3%91&crid=1651XJCJIY90X&dib=eyJ2IjoiMSJ9.z7VZ01yDMzS7FNoFVZYjfIIDIqJf7MWuKacqC0FVcexkbaaq2K3koWWNRLSjCUnNocgOZhSxSL_K2NLBBZrMZcpmaEX61JZhaHNEK6GLg-pEYKA2nXRwSKnqndWUS3hKDuTBenbCbF9ouzxqIJhS3vUE_hhOFrJp8Tlc9ngNTITlqc6fLt1BAs_ijswPUxupFI1jSGK1lUobc-yQ_mRvKlzIJFYo4byvBh0iwoiQtwk.e5isTzn0mwmTiPh46biH3ZfvGoMPDzyBZDeiYAUNsKQ&dib_tag=se&keywords=voltage+regulator+7809&qid=1749637707&sprefix=voltage+regulator+780%2Caps%2C158&sr=8-1-spons&sp_csd=d2lkZ2V0TmFtZT1zcF9hdGY&psc=1
Solid state Relay	1	2,20	2,20	https://www.amazon.com/-/es/HiLetgo-m%C3%B3dulo-estado-Control-fusible/dp/B00WSN9CJC/ref=sr_1_6?__mk_es_US=%C3%85M%C3%85%C5%BD%C3%95%C3%91&crid=1DCIOST74VCPI&dib=eyJ2IjoiMSJ9.q91PYm6OKAKVxQl4ebBf0AcV_wFfLUw6yFpImY74y9NNuAwuMA_hpIqRP2tM66p4flq29UABM9fA-Vt8GxLx1_6p2ie44_AJ84mtp5V95vCcb2_E7tkDR2bruMwsij8DGnkhkGYR3yGb3K202Yot5Uw3FOzQ5Em4ew4ZXLH8U5mn9tf4yMoJdQZ_yI1NPchNJZzmKsWA_Omo89YqgdAJFDpTr6nQ7RvzFNO0pdqxyZg.B-yP_CUBkbA0UKhlF3XE1GJNXnzZo4iaBUsoYxd5gl0&dib_tag=se&keywords=rele+solid+state+5V&qid=1749637984&sprefix=rele+solid+state+5v%2Caps%2C131&sr=8-6
Conectores	9	0,11	0,99	https://www.amazon.com/s?k=conectores+arduino&__mk_es_US=%C3%85M%C3%85%C5%BD%C3%95%C3%91&crid=ZOJJGKBHPROE&sprefix=conectores+arduino%2Caps%2C133&ref=nb_sb_noss
Capacitors	2	0,04	0,08	https://www.amazon.com/ALLECIN-surtido-condensadores-electrol%C3%ADticos-aluminio/dp/B0C1VBXCQM/ref=sr_1_1_sspa?__mk_es_US=%C3%85M%C3%85%C5%BD%C3%95%C3%91&crid=O5JYUB0HNKY2&dib=eyJ2IjoiMSJ9.ekYH7jx2EeGYfnpDMkTUbA5_8nAZM5NXRF7ISTCDYRdiGRRate4MlrQd9ldvZs-A_pzD-zjApyYWkhjoUOqNALpVcJsc6DD3JHlReRoBcxMOy5HnZgoPPDGKGQ7eKIroLYErlCkXUuGwYEHo_DbuP4dZ3WHupx5R5NgErb4ax20-0bVA56GPbf6AHFW0x2BVjzMCRqJcM72pPARxXIipVzthtF2eEvVnVx2oaxKJv7E.rax_WABdj5Kg3

				iZg0-DCwqXoqhX58FBhrXxgFDcFrrk&dib_tag=se&keywords=condensadores&qid=1749638632&sprefix=condensadore%2Caps%2C169&sr=8-1-spons&sp_csd=d2lkZ2V0TmFtZT1zcF9hdGY&psc=1
Arduino Nano	1	3,80	3,80	https://www.amazon.es/APKLVSR-Module-mega328P-Arduino-Puerto/dp/B0CLGX2FJ9/ref=sr_1_12?adgrpid=85854335224&dib=eyJ2IjoiMSJ9.wuytF6tMALHKEGy0GICqBwcRFQpKEZKCzqXad_AXyPJ0_ZDUmG2LH2KzV9ctFYWEWEEIUQdeLSri2Bg45C9iD2F49jhsRHYx0GSuxbxaEMBiPDua58xqp1x9_dKheBUPbNdEk-JjHMZgn76xIdAjKaPv5rarJb7O5lTwWRJcNRI5N3pF8bL31M7My_F3e9Md927yh0apMAnqn-Y1nYtAthjKEVgjyw-jpuIbzXAJIGMAug80pe5-bmORDw3LvE0VM7CZMo-7IsjBn-A7j7OBB-Gcl2gmtfvTs04erUCHXKc.hvT6x7Qzqxfu3LOOe9jiNlkBh-fmk7-1JV0vvlITVnk&dib_tag=se&hvadid=601256233111&hvdev=c&hvexpln=0&hvlocphy=9198606&hvnetw=g&hvocijid=13449499133394937419--&hvqmt=e&hvrand=13449499133394937419&hvtargid=kwd-360997649255&hydadcr=13835_2285390&keywords=arduino%2Bnano%2Bamazon&mcid=2524cbc3487437bd8c111f7168a8b433&qid=1759172850&sr=8-12&th=1
I2C LCD 1602 Module	1	6,48	6,48	https://www.amazon.es/Freenove-Display-Compatible-Arduino-Raspberry/dp/B0B76Z83Y4/ref=sr_1_5?__mk_es_ES=%C3%85M%C3%85%C5%BD%C3%95%C3%91&crid=2GV2SZOLXHTU8&dib=eyJ2IjoiMSJ9.zS7aZ6a2jGCbOuCGQBMdPBkJIp9SgLBVNvSsekQoKdSCrSsuhix_B_I3BC6wnefrEl-hT1g4GsH8P0PVqaE-apJblFfMOBwHr0wxDfmux65lhH5JkwSxi6x26Mbtf8i1J_kDUf4IxrmtkrSseUEYcvGkZ9asDAlG3kcetDrop6I60_ZHkz55nRl6JYLCa4v6gscZGa4xtwg3jEyuafU6IdebEaw-yGFjB2wIfa5ABxt9J7M6EWqmRzCOgz_x2qJfb162HNaJbpOofSgIbQWY3OGCPFNuDv-90fYz8gxFKKs.OHEfqWkwhTBIA3y2akaKxY_BA2xcuZvFExHS164B0aw&dib_tag=se&keywords=led%2Bscreen%2Barduino&qid=1759172906&sprefix=led%2Bscreen%2Barduino%2Caps%2C83&sr=8-5&th=1
Pump 60l/min	1	110,00	110,00	https://www.amazon.es/AquaForte-aluminio-Silenciosa-Capacidad-regulable/dp/B006SYHCI0/ref=sr_1_5?__mk_es_ES=%C3%85M%C3%85%C5%BD%C3%95%C3%91&crid=1Z9OHLAOKFU5L&dib=eyJ2IjoiMSJ9.NRkweEtlZU8--IlxdeJnBVnA57aO3bZv5hAEsbZEYcl9xBXcXFNMVAeKlU_86n4GJzWpJ4OIB5EVtu1BudyX06OpzDGO0m-QAcaWxxlnw97CIZDvWj0Q6BCLHxdqOjyrizC-5bgqUfTjGLc7AgJx9N7xc5UT6tWYHqJTycqoAMjZkBHAgosIT_-LUFZez5Z_CAMOPlR3IbjEVCJU9N_bvnvFdB68xiLwvSfY2hTUrhiUBE75D232bex3DA3G01LUZhVONH23IfRAz2U_0am5jKcT0EFB2WKabKOpAJOVNUM.SmnSqG7K3XQG0_bTTbTVbFH7hzXOCEkY8uRXlZar1lc&dib_tag=se&keywords=Bomba%2Bv30&qid=1749640500&sprefix=bomba%2Bv30%2Caps%2C130&sr=8-5&th=1
3D printing	-	Var	Variable	https://es.farnell.com/ultimaker/1609/filament-pla-black-750g/dp/2992628?gross_price=true&CMP=KNC-GES-GEN-SHOPPING-Pmax-High_ROAS&gad_source=1&gad_campaignid=18071281895&gbraid=0AAAAAD8yeHlKxiGMy79WCfJwNBT_BsqP9&gclid=Cj0KCQjw0qTCBhCmARIsAAj8C4b-IsF7T5oIuiyewkZV-nNr8tDFVX0Vy5St76HWuHN5j-02Z0rQICEaAhJBEALw_wcB
Led	1	0,05	0,05	https://www.amazon.es/BOJACK-iluminaci%C3%B3n-Componentes-componentes-electr%C3%B3nicos/dp/B07RTWQSJB/ref=sr_1_4_sspa?__mk_es_ES=%C3%85M%C3%85%C5%BD%C3%95%C3%91&crid=79LE5W27FDCU&dib=eyJ2IjoiMSJ9.7IvCTgIcejHAPAo0dMNXCOMj0-

				9ptJ75_i340cBNZe434WRnmQisZbledg5HWHcQmGdHyd5MKKYUofZIbiBn-h7oEPUayNQmbMIiEQoO5pb-BQUvo6ab9RjwgdN2j4-KIrzAPSk-d95b2uxX1MGxjABDvTRAANCTRzUDYq6MKZDjXNBtSsSuRY6rjOyL21JTPBXVeCmkfyMLuurMPP38dLma_jebtMaP3flJDMTmfREr7MVBV8Vy232o9s9cIB9F_qyfeTqPwCMznAyYafek-Kbum5npgzjXky_9h-sUmMY.s8mnD2I7y9azd-RIkPHHjDZx572i6GTF7-OJUjIJJ0E&dib_tag=se&keywords=led&qid=1759173028&sprefix=led%2B%2Caps%2C74&sr=8-4-spons&sp_csd=d2lkZ2V0TmFtZT1zcF9hdGY&th=1
Buzzer	1	1,40	1,40	https://www.amazon.es/ANGEEK-KY-012-Impresora-Raspberry-Unidades/dp/B07VR9441R/ref=sr_1_3_sspa?crid=1LGXO1020RK3M&dib=eyJ2IjoiMSJ9.Tu-AsHe4nFgT9ta-FHbumNZwgsrVjJirxH6HBdKA89eRkihHvGYrxhz6LWC7E7cS0j5RMRYHUQwvsKLjaO9UcwFYZh3PxrDCzOFWVsc2mq44XCsjttHasd1yI4RN8oMraayLyPRR0HkkPINKULgdBF6r_JeL8eGpsk9QkzLT0ApnmtIyvcZF__Ppu_quvpOJ-UbqG1fmv4KOaGH66SjsMfZjDk1ZUH2L4lKSqYAISI_Ck7V0VPzKBOMb1ftVW1rEC9Dz5LOgYOTNt-6I680GPMRo4fVydKmajsFGbbd2j1U.8dHzoC53_AmXh3nRmUExaqcDWaCQ3VNjJ28Yhbg2Q9k&dib_tag=se&keywords=buzzer+arduino&qid=1759173100&sprefix=buzzer+ardui%2Caps%2C75&sr=8-3-spons&sp_csd=d2lkZ2V0TmFtZT1zcF9hdGY&psc=1

The total cost of the materials for building the device is 248,72€ plus the cost of the 3D printed polymers. Materials such as resistors, LEDs, pin connectors, capacitors, PCB, ICs, and fuse holders were purchased as a kit, however, not all materials available in the set were used when building a single device. Most of them can be also easily reused from obsolete/damaged consumer electronic devices or household appliances.

3. Hardware Description

3.1. *3D Design and Printing*

The device enclosure was fabricated using 3D printing technology and has overall dimensions of x cm × x cm × x cm. The rear side of the case hosts the connectors for power supply, while the front panel integrates the main user interface elements, including the power switch, the LCD display for real-time visualization of sensor data, and the red alarm LED.

3.2. *Electronics*

The electronic system combines sensing, processing, and actuation components to ensure continuous monitoring and safety control within the chamber. Gas detection relies on two dedicated sensors connected via the I²C communication bus: an oxygen sensor (DFRobot Oxygen Sensor), which operates on an electrochemical principle to provide accurate and real-time O_2 concentration measurements in the range of 0–25% with 0.1% resolution and long-term stability, and a carbon dioxide sensor (Sensirion SCD4x series), a compact device based on photoacoustic spectroscopy capable of detecting CO_2 concentrations between 400 ppm and 5000 ppm with high precision and low power consumption. The LCD display, also interfaced through I²C, continuously reports the measured values and system status to the user.

Data acquisition and control are managed by an Arduino Nano microcontroller, which processes sensor readings and governs the actuators. Whenever one of the monitored gases exceeds the predefined thresholds, the microcontroller activates the ventilation pump through a solid-state relay connected directly to its digital output. In addition, the system integrates a alarm mechanism consisting of a red LED positioned on the enclosure front panel, which flashes, and a buzzer that generates an acoustic signal, until environmental parameters return to safe ranges.

3.3. *Arduino control*

At startup, all peripherals are initialized, including the CO_2 and O_2 sensors, the LCD module, and the actuators such as the ventilation pump, the buzzer, and the alarm LED. The display provides immediate feedback on the system status and, once initialization is complete, it continuously shows the gas concentrations in real time.

During operation, the microcontroller periodically retrieves measurements from the CO_2 sensor, which can also provide temperature and humidity data, and from the O_2 sensor, ensuring that both gas concentrations are constantly monitored. The acquired values are compared against predefined safety thresholds, with upper limits set for CO_2 and lower limits for O_2. Whenever one of these thresholds is exceeded, the system enters an alarm state: the ventilation pump is activated to restore optimal conditions inside the chamber, the buzzer and external LED are triggered in a blinking mode to provide both acoustic and visual alerts, and the LCD display replaces the normal readout with explicit warning messages highlighting the parameter that caused the alarm.

The firmware also integrates a recovery mechanism that prevents oscillations between alarm and non-alarm states. Once the monitored values return within safe ranges, the system automatically deactivates the alarms, switches off the pump, and restores the standard visualization on the display. In this way, the firmware ensures continuous supervision of the chamber parameters and immediate intervention in case of non-optimal conditions.
