# Supplementary material for: Optimized Open-Source Setting for Subjecting Rodents to Chronic Normobaric Hypoxia in Facilities with Minimal Nitrogen Supply
Source: Life (Basel). 2026 Jul 9;16(7):1140. doi: 10.3390/life16071140 (PMC13412489; doi:10.3390/life16071140)

1. **CO_2 variability within the hypoxia chamber.**

CO_2 concentration measured at 9 different mice cages distributed within the hypoxic chamber. See main text for explanation.

**Cage position**	**CO_2 (ppm)**
1	928
2	972
3	935
4	877
5	919
6	975
7	838
8	862
9	841
mean	905
SD	53
**CV (%)**	**5.82**

---

2. **Change in O_2 concentration within the hypoxia chamber after opening and closing one of the doors for extrating or placing one of the mice cages**

Example recorded in one of the maneuvers. See main text for explanation.

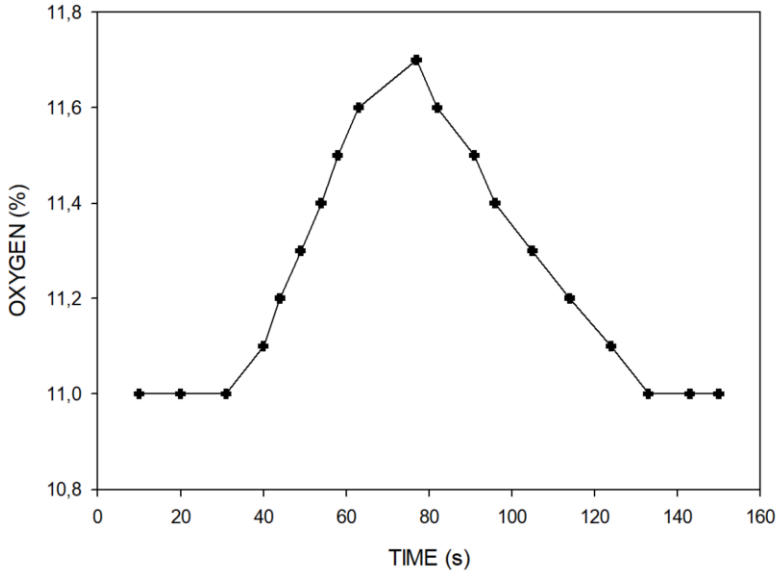

Supplement: Supplementary file 1 [file life-16-01140-s001.zip › Supplementary results.pdf]
