# Supplementary figures and images for: Optimized Open-Source Setting for Subjecting Rodents to Chronic Normobaric Hypoxia in Facilities with Minimal Nitrogen Supply
Source: Life (Basel). 2026 Jul 9;16(7):1140. doi: 10.3390/life16071140 (PMC13412489; doi:10.3390/life16071140)

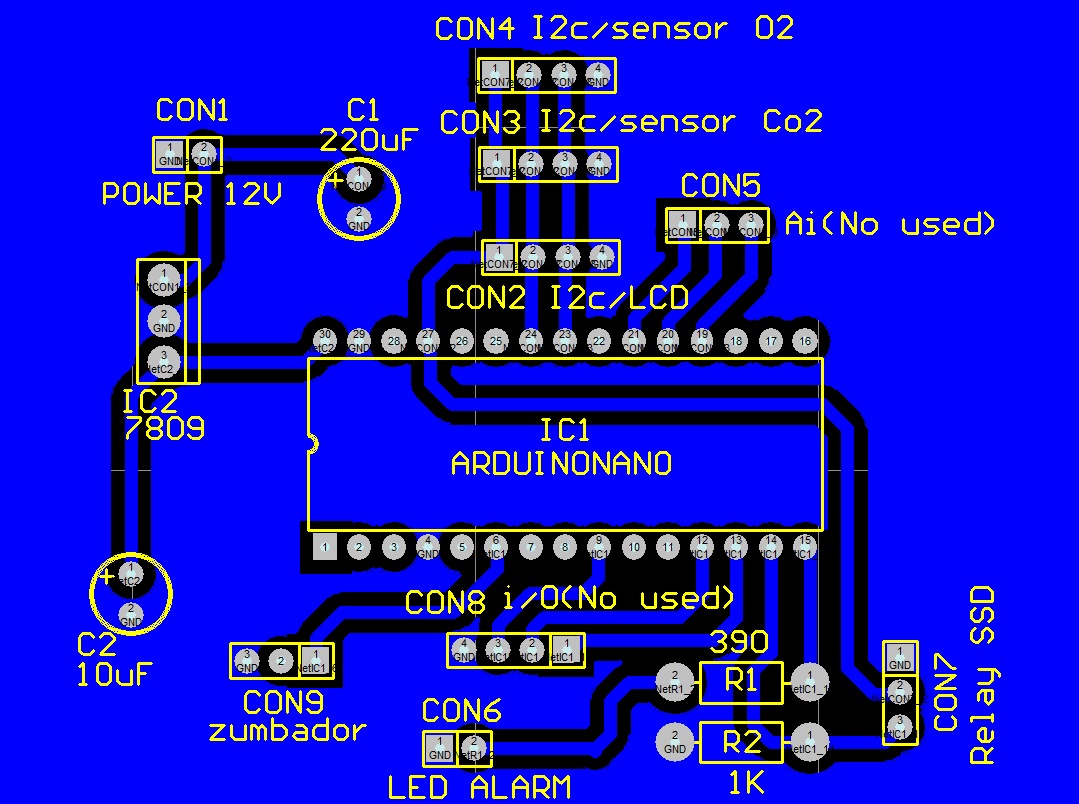

Supplement: Supplementary file 1 [file life-16-01140-s001.zip › FailSafe/PCB/pcb_alarma.jpg]

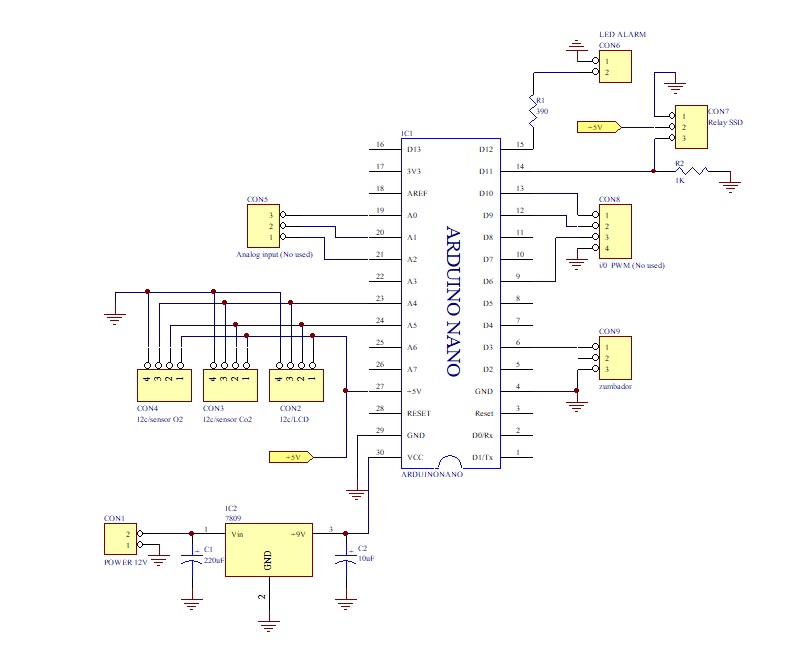

Supplement: Supplementary file 1 [file life-16-01140-s001.zip › FailSafe/PCB/schematic.jpg]

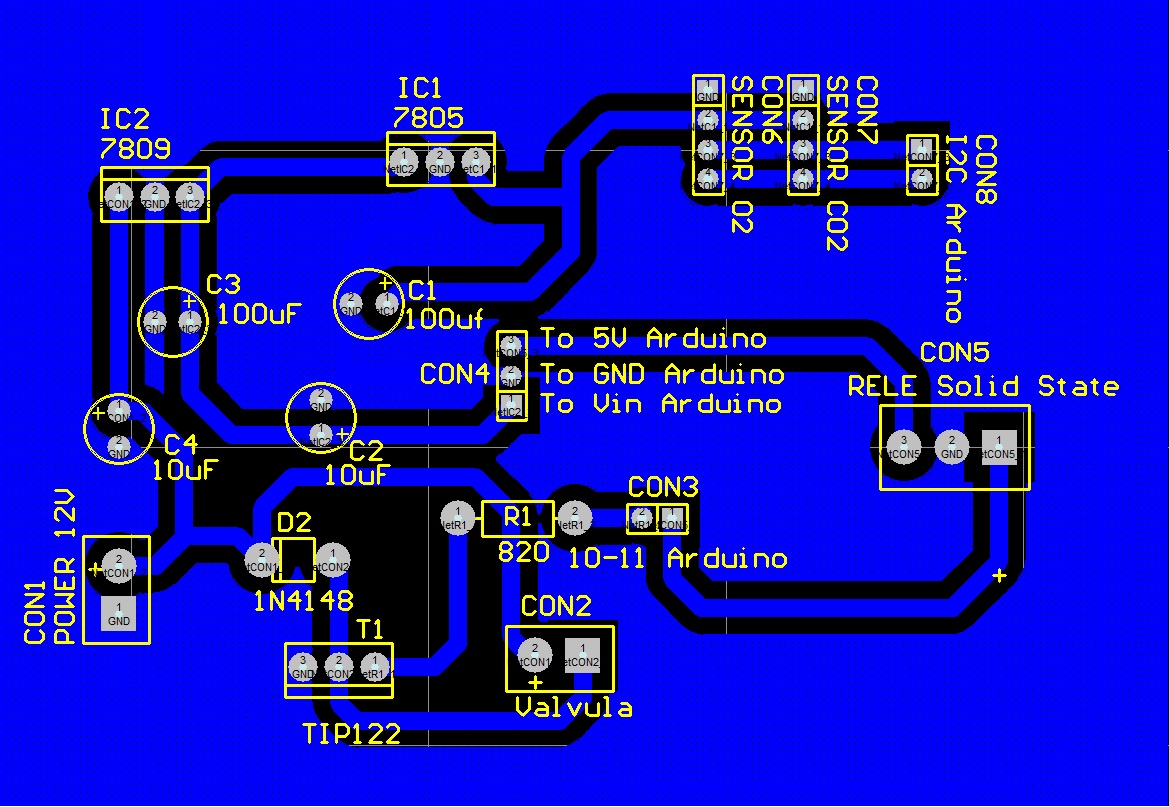

Supplement: Supplementary file 1 [file life-16-01140-s001.zip › PCB/PCB.jpg]

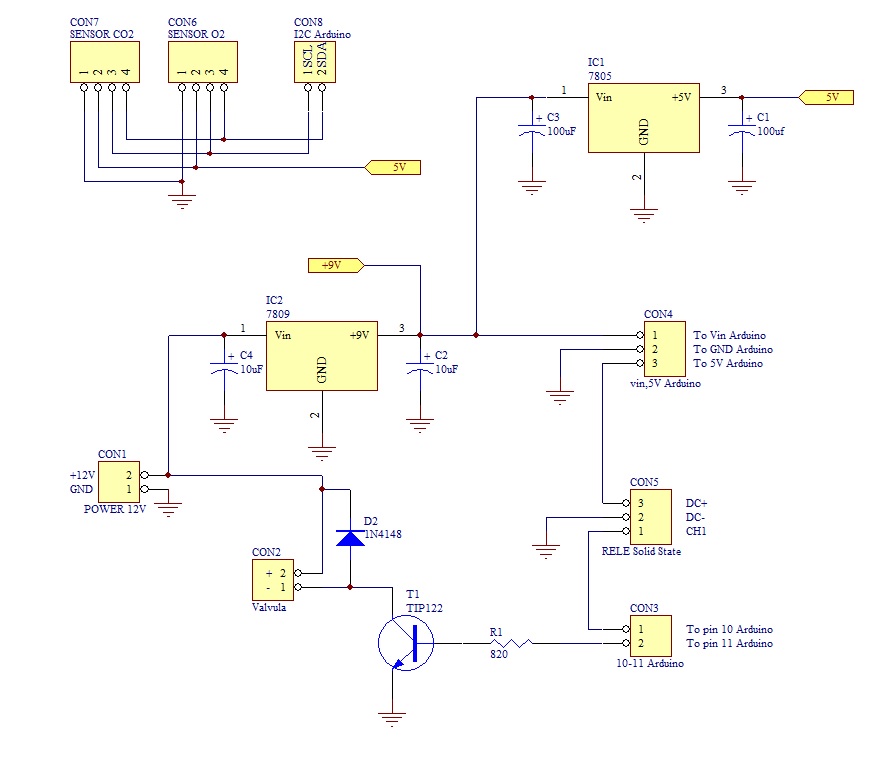

Supplement: Supplementary file 1 [file life-16-01140-s001.zip › PCB/sch.jpg]
